# Phenotypic and histochemical traits of the interaction between *Plasmopara viticola* and resistant or susceptible grapevine varieties

**DOI:** 10.1186/1471-2229-12-124

**Published:** 2012-08-01

**Authors:** Silvia Laura Toffolatti, Giovanni Venturini, Dario Maffi, Annamaria Vercesi

**Affiliations:** 1 Dipartimento di Scienze Agrarie e Ambientali - Produzione, Territorio, Agroenergia (DiSAA), Università degli Studi di Milano, via Celoria 2, 20133, Milan, Italy

**Keywords:** Disease resistance, Oomycetes, Pathogen fitness

## Abstract

**Background:**

Grapevine downy mildew, caused by *Plasmopara viticola*, is a very serious disease affecting mainly *Vitis vinifera* cultivated varieties around the world. Breeding for resistance through the crossing with less susceptible species is one of the possible means to reduce the disease incidence and the application of fungicides. The hybrid Bianca and some of its siblings are considered very promising but their resistance level can vary depending on the pathogen strain. Moreover, virulent strains characterized by high fitness can represent a potential threat to the hybrid cultivation.

**Results:**

The host response and the pathogen virulence were quantitatively assessed by artificially inoculating cv Chardonnay, cv Bianca and their siblings with *P. viticola* isolates derived from single germinating oospores collected in various Italian viticultural areas. The host phenotypes were classified as susceptible, intermediate and resistant, according to the Area Under the Disease Progress Curve caused by the inoculated strain. Host responses in cv Bianca and its siblings significantly varied depending on the *P. viticola* isolates, which in turn differed in their virulence levels. The fitness of the most virulent strain did not significantly vary on the different hybrids including Bianca in comparison with the susceptible cv Chardonnay, suggesting that no costs are associated with virulence. Among the individual fitness components, only sporangia production was significantly reduced in cv Bianca and in some hybrids. Comparative histological analysis revealed differences between susceptible and resistant plants in the pathogen diffusion and cytology from 48 h after inoculation onwards. Defence mechanisms included callose depositions in the infected stomata, increase in peroxidase activity, synthesis of phenolic compounds and flavonoids and the necrosis of stomata and cells immediately surrounding the point of invasion and determined alterations in the size of the infected areas and in the number of sporangia differentiated.

**Conclusions:**

Some hybrids were able to maintain an intermediate-resistant behaviour even when inoculated with the most virulent strain. Such hybrids should be considered for further field trials.

## Background

*Plasmopara viticola* (Berk. *et* Curt.) Berlese and De Toni is an obligate parasite, able to biotrophically grow inside the susceptible tissues of species belonging to the genus *Vitis* and particularly of the extremely susceptible *Vitis vinifera* L., which is, unfortunately, the only species extensively used in the global wine industry [1]. *P. viticola* infects all green parts of the vine [2], leaves and bunches in particular, penetrating through stomata and extensively forming an intercellular mycelium with haustoria in the mesophyll cells [3]. In favourable climatic conditions, the pathogen causes numerous infection cycles, which are responsible for both quantitative and qualitative yield reductions. Since agricultural practices are almost ineffective in reducing disease incidence [4], chemical control is, at the moment, the most efficient mean to prevent severe downy mildew epidemics [5] and obtain good quality grapes.

Damages due to *P. viticola* could be reduced by using less susceptible grapevine varieties obtained by crossing *V. vinifera* with resistant species and in particular with American *Vitaceae* co-evolved with the pathogen such as *V. labrusca* L., *V. aestivalis* Michx., *V. rupestris* Scheele and *V. riparia* Michx. [6]. The resulting hybrids, however, are often unsuitable for the production of high quality wines due to their unpleasant flavours and aromas.

Both monogenic and polygenic resistance to *P. viticola* have been described in the *Vitaceae* family [7,8]. More recently, single quantitative trait loci (QTL) have been associated with resistance to *P. viticola* in *V. amurensis* Ruprecht [9], *V. riparia*[10] and in *V. vinifera* cv Regent [11]. Monogenic resistance is determined by a single dominant gene and has been associated with an hypersensitive response causing stomatal death at the point of infection and was found in the genera *Tetrastigma* and *Cissus*[7], whereas polygenic resistance induces a reduction of the pathogen growth after infection and is polygenically determined. The resistance imposed by a single gene is usually easily circumvented by pathogens characterized by a great evolutionary potential [12] such as *P. viticola* which has a high asexual sporulation efficiency, a polycyclic behaviour and sexual reproduction through the formation of oospores. However, also in polygenic resistance, that leads to a disease reduction rather than the absence of disease, host genotypes can respond differently to different pathogen strains [13] upon pathogen adaptation [14]. In the coevolutionary arms race between host and pathogen, the pathogen possesses the advantage of relatively short generation times and higher abundance in number compared to the host [15]. As a consequence, the pathogen can overcome the host defence mechanisms.

The variety Bianca, achieved by crossing the susceptible *V. vinifera* cv Bouvier with the resistant interspecific hybrid Villard Blanc [16], is together with Regent and Solaris one of the few cultivated hybrids resistant to *P. viticola.* Due to the multiple crosses involved in the breeding program, the genetic background of Bianca is mainly composed by *V. vinifera* (78.09%), and also by the Northern American species *V. rupestris* (14.58%), *V. berlandieri* Planch. (3.13%), *V. lincecumii* Buckl. (2.64%) and *V. labrusca* (1.56%) [17]. Recently, QTL mapping showed that resistance in cv Bianca is controlled by a single dominant allele at the *Rpv3* locus on chr18, that explains most of the phenotypic variance in downy mildew resistance [18]*.* The resistance phenotypic traits observed in Bianca are localized necrosis and reduction of the colonization extent and sporulation capacity of *P. viticola*. Information on the interactions between *P. viticola*, cv Bianca and its siblings obtained from a cross with Chardonnay mainly derives from experimental inoculations carried out with isolates coming from naturally infected leaves collected in vineyards [18-20]. Since in single leaves different *P. viticola* genotypes can coexist, host reactions can not be attributed to a specific interaction with a pathogen individual. Therefore both the possible range and effectiveness of resistance should be ascertained only by using single spore isolates, i.e. distinct pathogen genotypes, and by quantitatively assessing disease parameters such as the area under disease progress curve (AUDPC). Host responses to individual *P. viticola* genotypes provide not only a profile of resistance but also insights on the evolutionary potential of the pathogen strains through the investigation of their virulence, defined as degree of damage caused to the host [21], and their fitness *i.e.*their ability of surviving and reproducing [22]. A rapid increase of genotypes characterized by the ability to breakdown monogenic/polygenic resistance, as already assessed on cv Bianca in Czech Republic where it is cultivated on a limited acreage [19], affects both effectiveness and durability of resistance [13].

Detailed investigations carried out on various host-pathogen binomials pointed out that resistance reactions to biotrophic fungal infections depend on the activation of several defence mechanisms, including the production of antimicrobial metabolites and proteins (pathogenesis related proteins, PRs) and, at the cell wall level, thickenings, callose appositions in the paramural space and accumulation of phenolic compounds and reactive oxygen species [23,24]. While defence reactions against *P. viticola* have been investigated at the morphological and histochemical level mainly on resistant species and only on two resistant hybrids, Solaris [25] and Seyval [26], incomplete information on the resistance mechanisms and on their histochemical features are available on cv Bianca and its siblings. Moreover, since the pathogen colonization pattern and the host histochemical reactions have often been independently examined, it is not clear if the observed structural or histochemical changes in the grapevine tissues are actually associated with the pathogen presence.

The aims of the present work are: (i) to quantitatively evaluate the phenotypic responses of both the parental and the hybrid lines obtained by crossing the susceptible cv ‘Chardonnay’x‘Bianca’ to 5 *P. viticola* strains each of which derived from a single oospore and assess both the host resistance level and the virulence of the pathogen isolates; (ii) to estimate the fitness of the most virulent isolate of *P. viticola*; (iii) to characterize at different infection stages the histochemical reactions of the host plants associated with the colonizing structures of the most virulent strain of *P. viticola.*

## Results and discussion

### Evaluation of the disease severity

The first sporangia differentiated by *P. viticola* were generally observed 5–6 days after inoculation. The percentage of sporulating leaf discs did not show great variations, being in most of the cases higher than 50%, whereas variable values of PSA, ranging from 0 to 69%, characterized the different grapevine lines inoculated with *P. viticola* strains (data not shown).

#### Comparison among strains

Since significant interactions were found among isolates and cv/hybrids (Table 1), strain virulence was estimated on each cv/hybrid individually. Except for Chardonnay and the hybrids 18017, 18036 and 18096 (df 4, 10; F < 2.6; *P* > 0.05), AUDPC (Table 2) significantly varied among the isolates on the different hybrids (df 4, 10; F > 3.7; *P* < 0.05). In general, G was the most virulent isolate (mean AUDPC 106.2), followed by BAI (78.1), MIX (69.6) and C (62.7), whereas SO showed lower virulence (37.3).

**Table 1:** Two-factors ANOVA for disease symptoms expressed as AUDPC

**Variable**	**Type III sum of squares**	**df**	**Mean sum of squares**	**F**	**Sig.**
Model	648397.3	84	7719.0	6.11	1.07 × 10^-23^
Intercept	1277882.8	1	1277882.8	1011.63	1.76 × 10^-73^
cv/hybrid	212910.0	16	13306.9	10.53	2.69 × 10^-18^
Isolate	127372.1	4	31843.0	25.21	2.1 × 10^-16^
cv/hybrid x isolate	308115.2	64	4814.3	3.81	2.09 × 10^-12^
Error	214743.1	170	1263.2		
Total	2141023.2	255			

**Table 2:** **AUDPC values and derived phenotype (P) of different cv/hybrids inoculated with each *P. viticola *strain**

**cv/hybrid**	**BAI**		**SO**		**C**		**G**		**M**		**Overall**
	**AUDPC**	**P**	**AUDPC**	**P**	**AUDPC**	**P**	**AUDPC**	**P**	**AUDPC**	**P**	**phenotype**
Chardonnay	106.5 cdef	I	85.7 cd	S	46.4 abc	I	80.3 abcd	I	66.1 abcde	I	IS
Bianca	7.7 a	R	1.8 ab	R	50.0 abc	I	108.9 abcdef	I	71.4 abcde	I	IR
18001	32.8 bcd	I	1.8 ab	R	0 a	R	184.5 ef	S	83.9 abcdef	I	V
18006	119.0 def	S	42.9 abcd	I	75 abc	I	156.6 def	S	36.3 abc	I	IS
18017	112.5 ef	S	48.2 abcd	I	85.7 abcd	I	114.9 abcdef	I	73.8 abcde	I	IS
18036	10.7 ab	R	55.4 abcd	I	50.0 abc	I	47.0 ab	R	48.8 abcde	I	IR
18048	191.1 f	S	44.7 abcd	I	151.8 cd	S	154.2 cdef	I	134.5 def	S	IS
18053	83.3 cdef	I	123.2 d	S	69.6 abc	I	108.9 abcdef	I	31.0 abc	I	IS
18088	53.0 cdef	I	60.7 bcd	I	71.4 abc	I	187.5 f	S	181.6 f	S	IS
18090	119.6 ef	S	0 a	R	100 bcd	I	142.9 bcdef	I	35.7 abc	I	V
18096	70.2 cdef	I	12.5 abc	I	8.9 ab	R	46.4 a	R	38.7 abcd	I	IR
18099	38.7 bcde	I	48.2 abcd	I	73.2 abc	I	57.7 abc	R	1.2 ab	R	IR
18100	114.9 ef	S	48.2 abcd	I	55.4 abc	I	42.9 a	R	108.9 bcdef	I	V
18103	51.2 cdef	I	46.4 abcd	I	50 abc	I	95.2 abcde	I	3.6 ab	R	IR
18106	106.6 def	S	10.7 abc	I	10.7 ab	R	90.5 abcde	I	117.8 cdef	S	V
18110	81.0 cdef	I	0 a	R	167.9 d	S	122.6 abcdef	I	150.6 ef	S	V
18120	28.5 bc	R	3.6 abc	I	0 a	R	64.9 abcd	I	0 a	R	IR

Based on the results of ANOVA and multiple comparison of the means, the plant individual phenotypes are classified as susceptible (S), intermediate (I) or resistant (R). Overall phenotypes of each cv/hybrid are intermediate-susceptible (IS), intermediate-resistant (IR) or variable (V). Mean values within the same column followed by the same letter are not significantly different at the *P* = 0.05 level of probability.

#### Comparison among cv/hybrid lines

Considering each *P. viticola* strain, significant differences (df 16, 34; 4.3 < F < 9.7; *P* = 0.000) among the AUDPC values observed on the different cv/hybrids have been found (Table 2). Based on statistical analysis, host plants have been classified in three different phenotypes: resistant, characterized by low AUDPC; susceptible, with high AUDPC; and intermediate, with AUDPC values not significantly different from the previous ones. For example, following the inoculation with BAI isolate, the parental hybrid Bianca and two offsprings (18036 and 18120) showed a resistant behaviour, 6 offprings (18006, 18017, 18048, 18090, 18100 and 18106) were susceptible, and the remaining ones intermediate (Table 2). As shown in Table 2, the classification of the host plants varied depending on the inoculated strain: for instance, hybrid 18001 was susceptible to strain G, resistant to SO and C and intermediate with BAI and M. Interestingly, the two parents, Chardonnay and Bianca, showed clearly distinct phenotypes, susceptible and resistant, when inoculated with BAI and SO isolates, and an analogous intermediate behaviour when challenged with the remaining three strains. Therefore, among the five isolates tested, three strains induced similar AUDPC in both parental lines showing that resistance mechanisms present in Bianca were not effective in limiting their growth. Overall, Chardonnay and 5 offsprings (18006, 18017, 18048, 18053 and 18088) can be considered intermediate-susceptible, Bianca and 5 offsprings (18036, 18096, 18099, 18103 and 18120) intermediate-resistant, while the remaining hybrids (18001, 18090, 18100, 18106 and 18110) show a variable phenotype (susceptible, resistant or intermediate) depending on the pathogen isolate used for the inoculation.

The significant host/pathogen interaction suggests that different host phenotypes correspond to different pathogen genotypes. However, in most of the cases the plants showed an intermediate-susceptible or intermediate-resistant phenotype. The most interesting hybrid found in this study, 18120, resulted resistant to three strains, BAI, C and M, and intermediate to the most and the least virulent strains, G and SO. However, once the resistant cv is cultivated, strains able to colonize resistant grapevine accessions could cause severe damages only if they are characterized by high fitness levels.

#### Pathogen fitness

According to Kruskal-Wallis test, the fitness components of the most virulent *P. viticola* strain (G) varied among the different grapevine lines (df:16; H 37.51, sig. 0.02 for T_10_; H 36.51, sig. 0.02 for MGR; H 34.36, sig. 0.005 for IEI; H 42.85, sig. 0.000 for SPOR). About 30% of the grapevine lines considered in this study induced low values of the fitness parameters considered, 30% high values and the remaining ones an intermediate behaviour (Table 3). In the considered experimental conditions, 10% of the leaf was covered by sporangia in about 6–7 days, the maximum absolute rate of disease increase occurred two days later and infection efficiency, estimated 10 days after inoculation, was about 0.5. Apart from SPOR, that was particularly high and associated with a wide infected area, the fitness of *P. viticola* on cv Chardonnay was generally intermediate. The highest PF, associated with particularly low T_10_ and MGR and high IEI, was found on the hybrids 18001 and 18088. On the resistant parental Bianca, the pathogen was characterized by low T_10_, MGR and high IEI, therefore by a rapid and widespread colonization of the tissues, but reduced SPOR and, as a consequence, the pathogen showed an intermediate PF. As indicated by T_10_ and MGR, which are estimates of the time required for disease severity to reach 10% and the inflection point respectively, the growth of the pathogen was slower on 18096, 18100 and 18120. On these lines IEI was moreover restricted, but while the number of sporangia differentiated per unit of infected area was reduced on 18096, significantly higher values of SPOR, analogous to those of the susceptible parental line Chardonnay, were detected on 18120. No differences were observed in the germination rates of sporangia differentiated in the different *Vitis* genotypes.

**Table 3:** Estimated components of fitness and composite fitness index of strain G on each cv/hybrid

**cv/hybrid**	**T**_**10**_	**MGR**	**IEI**	**SPOR**	**PF**
Chardonnay	170.58 abcdefg	9.20 abcdefg	0.49 abcde	12.05 ghi	3.80 abcdefg
Bianca	143.65 abc	8.63 abc	0.53 cde	10.13 abcd	4.98 cdefg
18001	123.19 a	7.09 a	0.75 e	11.12 defg	9.63 g
18006	132.89 ab	8.70 abcde	0.50 bcde	9.91 abc	4.40 defg
18017	145.20 abcd	8.72 abcdef	0.51 cde	9.83 ab	4.11 bcdefg
18036	183.17 cdefg	12.51 defg	0.27 abc	11.25 defgh	1.03 abcd
18048	131.72 ab	8.65 abcd	0.49 abcde	10.88 abcdef	4.93 efg
18053	177.97 bcdefg	12.74 efg	0.23 ab	10.94 bcdef	1.20 abcde
18088	102.66 a	6.85 ab	0.68 de	10.96 cdef	16.57 g
18090	149.80 abcde	8.35 abc	0.64 de	11.31 efgh	6.77 fg
18096	301.98 g	22.67 g	0.16 a	9.93 ab	0.33 a
18099	193.15 defg	11.38 bcdefg	0.26 abcd	10.26 abcde	1.34 abcdef
18100	211.01 efg	13.39 fg	0.24 abc	9.09 a	0.66 abc
18103	187.59 cdefg	12.02 cdefg	0.27 abcde	12.04 hi	1.86 abcdefg
18106	190.09 cdefg	12.12 abcdefg	0.32 abcde	13.93 i	2.69 abcdefg
18110	154.76 abcdef	8.46 abc	0.61 de	11.34 efghi	5.75 fg
18120	231.02 fg	15.67 g	0.12 a	12.05 fghi	0.46 ab

The individual fitness components are T_10_, MGR, IEI and SPOR, the composite index is PF. Mean values within the same column followed by the same letter are not significantly different at *P* = 0.05 level of probability.

The most virulent strain, G, showed an analogous composite fitness index on the susceptible and resistant parental cv, Chardonnay and Bianca. Since fitness can be defined as the combined ability of an organism to survive and reproduce, it seems likely that no costs to the pathogen are associated with its capacity to overcome the host resistance mechanism. Despite the absence of significant differences in the composite fitness index, it must be pointed out that in absolute terms on cv Chardonnay and in general on the IS hybrids, the pathogen more extensively colonized the tissues and differentiated an higher number of sporangia than on cv Bianca and other IR hybrids, such as 18120.

### Histological analyses

#### Colonization pattern

**Figure 1:**
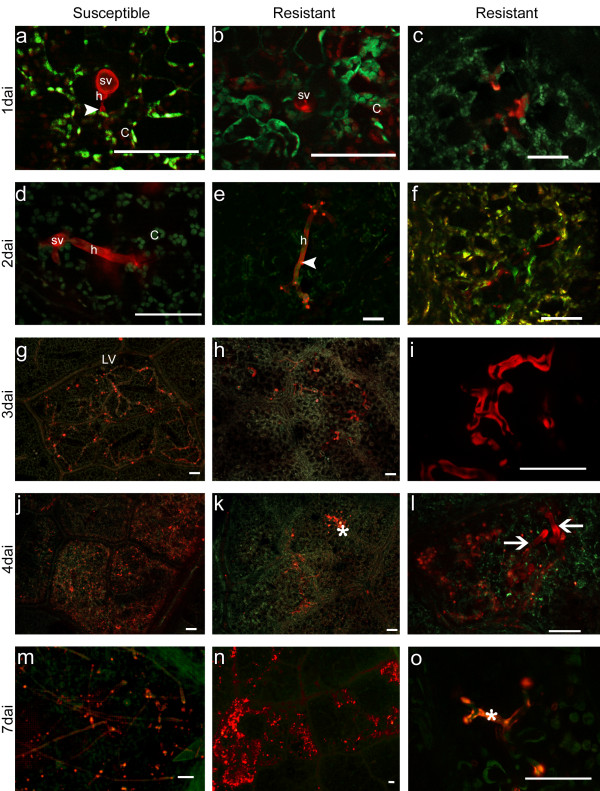
**Time course of colonization of grapevine cv/hybrids by *P. viticola *strain G.** The pathogen structures (red colour) inside the leaf tissues (green colour) of susceptible (a, d, g, j, m) and resistant (b, c, e, f, h, i, k, l, n, o) grapevine plants following inoculation with G strain and aniline blue staining are represented. Pathogen diffusion was monitored 1 (a-c), 2 (d-f), 3 (g-i), 4 (j-l) and 7 (m-o) days after inoculation. (**a**) Substomatal vesicle (sv) with primary hypha (h) and first haustorium (harrow head) in the substomatal cavity of the leaf tissues of hybrid 18006. (**b**) Substomatal vesicle under an infected stoma of grapevine line 18096. (**c**) Callose depositions (asterisks) on the spongy mesophyll cells under an infected stoma. (**d,e**) Branched hyphae with haustoria (arrowhead) developing from the substomatal vesicles inside susceptible cv Chardonnay and resistant hybrid 18120. (**f)** Low diameter hypha (arrow) in the resistant cv Bianca. (**g**) Regular diffusion of the mycelium inside an infected area of Chardonnay. (**h**) Reduced colonization of the leaf tissues of the resistant hybrid 18103. (**i**) Callose on the hyphal wall inside the resistant hybrid 18120. (**j**) Complete colonization of the infected areas of cv Chardonnay. (**k**) Limited diffusion of *P. viticola* mycelium that in some cases was covered by callose (asterisk). (**l**) Degenerated mycelium (left) under a necrotic area and regular hyphae (arrows) inside the same infected leaf of the intermediate hybrid 18090. (**m**) Sporangiophores emerging from Chardonnay. (**n**) Mycelium diffusion inside Bianca. (**o**) Detail of an hypha covered by callose (asterisk). C = chloroplast, LV = leaf vein. Scale bar = 50 μm.

**Figure 2:**
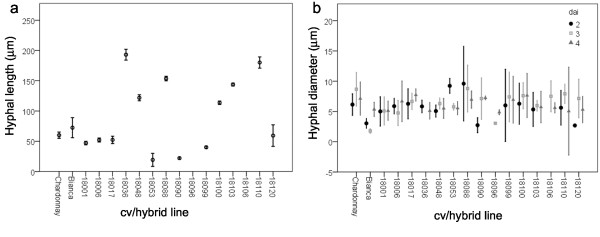
**Early development of *P. viticola *inside the leaf tissues.** Hyphal length (**a**) and diameter (**b**) measured at 2 and 2–4 dai respectively. Bars represent confidence interval (95%).

Encysted and germinating zoospores were observed in proximity of the stomata 24 hours after inoculation on all the examined grapevine lines. In most of the cases the substomatal vesicle, originating from the infection peg, was visible (Figure 1b), whereas just in a few infected stomata the primary hyphae with the first haustorium were also recognizable (Figure 1a). In some cases (18103 and 18110), callose depositions were observed on the cells surrounding the substomatal cavity immediately underneath the germinating zoospore (Figure 1c). At 2 dai, the primary hypha with haustoria, recognizable from the brightly fluorescent neck covered by callose, started branching inside the leaf tissues of all the plants except for 18096 and 18106 (Figure 1d-f). However, some differences in the pathogen structures could be detected. Due to the great variability observed in the length of the primary hypha, the different cv/hybrids could be divided into three groups: the first characterized by high values, ranging between 100–200 μm, of the pathogen vegetative structures, the second by an intermediate range (45–100 μm) and the third by low values (44–19 μm). Members of the different resistant classes could be found in each group (Figure 2). For example, the first group includes 18048, 18088 (IS), 18036, 18103 (IR), 18100 and 18110 (V), while the second contains the parental cultivars and 18006, 18017 (IS), 18120 (IR) and 18001 (V) and the third 18053 (IS), 18099 (IR) and 18090 (V). Due to the extensive growth inside the tissues of some grapevine plants, the hyphal growth could not be measured from 3 dai onwards. The hyphal diameter ranged between 5 and 10 μm on most of the investigated cv/hybrids except for a few cases including the IR Bianca at 2 and 3 dai (3 and 1.8 μm), 18120 at 2 dai (2.7 μm) and 18096 at 3 dai (3.1 μm) and the V 18090 at 2 dai (2.7 μm).

**Figure 3:**
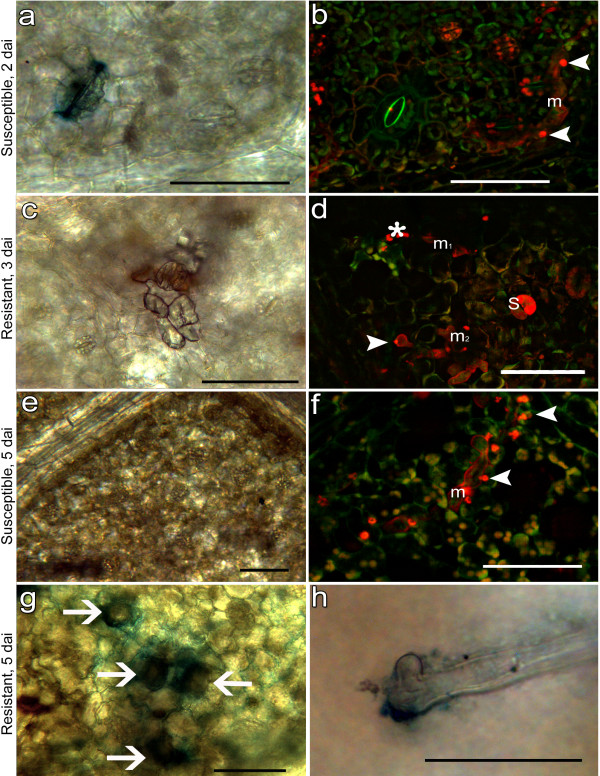
**Localization of peroxidase acitivity in infected leaf tissues.** POX (blue) have been visualized by transmission light microscopy following TMB staining (a, c, e, g, h). The corresponding *P. viticola* vegetative structures (red) inside the leaf tissues (green) have been visualized in epifluorescence after aniline blue staining (b, d, f). (**a**) Stoma of the susceptible hybrid 18048 showing POX at 2 dai. (**b**) *P. viticola* mycelium (m) with haustoria (arrowhead) close to the stoma visualized in (a). Autofluorescent red chloroplasts inside the stomata are visible. The picture was obtained by overlapping a surface section (epidermal cells) and a lower section (mesophyll). (**c**) Epidermal cells of the resistant cv Bianca showing POX on the plasma membrane, that is detached from the wall, and browning under the stoma at 3 dai. (**d**) Lower section of (c) showing an altered mycelium (m_1_) intensely coloured, probably due to callose deposition (asterisk), and no haustoria immediately under the browning area and regular vegetative structures of the pathogen (m_2_) in a surrounding area showing no peroxidase activity. Asterisk indicates callose, S callose in a closed stoma. (**e**) Leaf tissues of cv 18048 lacking POX at 5 dai. (**f**) Regular mycelium (m) with haustoria (arrowheads) in the same area. (**g**) POX in the epidermal walls of the cells surrounding the stomata with sporangiophores (arrows). Hybrid 18100, 5 dai. (**h**) Detail of a sporangiophore with POX at the exit point. Scale bar = 50 μm.

At 3 dai the differences between susceptible and resistant plants were clearly visible. While in the first case *P. viticola* started spreading inside the infected tissues and was mainly limited by the leaf veins (Figure 1g), in the resistant individuals the mycelium growth was restricted to the area close to the penetration stoma (Figure 1h) and in some cases brightly fluorescent appositions of callose were observed around the hyphae (Figure 1i,o) and in the surrounding stomata (Figure 3d). At 4 dai, the mycelium diffusion inside the susceptible individuals almost completely covered the infected area (Figure 1j), while on the resistant ones the pathogen growth was still restricted (Figure 1k) and degenerated hyphae were visible near the penetration point. However, close to the collapsed pathogen structures, it was still possible to observe some normal hyphae characterized by regular diameters (Figure 1l). At 7 dai the mycelium growth increased (Figure 1n) and sporulation occurred (Figure 1m) in all the samples even if at a variable rate.

**Figure 4:**
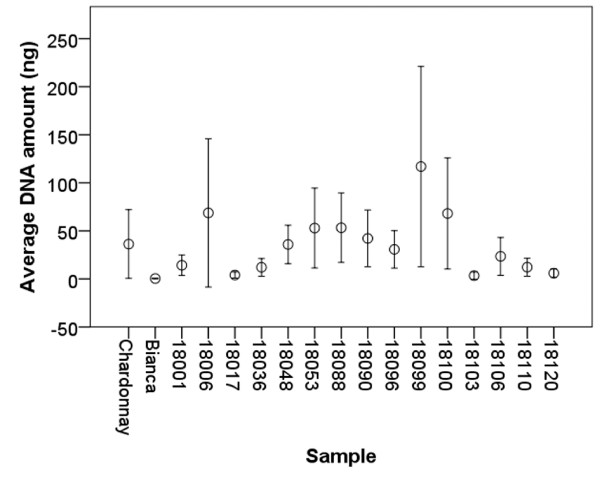
**Average *P. viticola *DNA amount (ng) in the leaf discs.** Bars represent confidence interval (95%).

The amount of *P. viticola* DNA inside each of the 12 inoculated leaf discs was estimated quantitatively by real-time PCR (calibration curve: y = −3.4x + 25.4; r^2^ = 0.996; PCR efficiency = 0.97). The average DNA amount ranged between 20–70 ng/leaf discs in most of the samples apart from Bianca, 18001, 18036, 18103, 18110, 18120, showing lower values (0.5-14.2 ng), and 18099, characterized by a higher value (117 ng) (Figure 4). A positive linear correlation was found among the amount of *P. viticola* DNA, the dimension of the infected areas and the number of sporangia differentiated by the pathogen on the susceptible parental line Chardonnay, suggesting that a high hyphal density and an abundant sporulation correspond to an extensive mycelium colonization (Table 4). A positive correlation between the dimension of the infected areas and sporulation was also found on the resistant cv Bianca. However, a negative correlation characterized the amount of *P. viticola* DNA and both dimension of the colonized areas and amount of sporangia, suggesting that in this case growth and sporulation are associated with a low hyphal density. In most of the hybrids examined, no significant correlation was found among the parameters except for 18036 (DNA-infected areas), 18053 (DNA-sporangia) and 18106 (infected areas-sporangia).

**Table 4:** Correlation among DNA amount, infected area and number of sporangia

**Cv/hybrid**	**Variable**	**Infected area**	**Sporangia**
Chardonnay	DNA	0.55 (0.07)	0.700 (0.01)
	Infected area	1	0.721 (0.01)
Bianca	DNA	−0.811 (0.001)	−0.748 (0.005)
	Infected area	1	0.723 (0.008)
18036	DNA	0.664 (0.018)	-
	Infected area	1	-
18053	DNA	-	0.599 (0.04)
	Infected area	1	-

Spearman’s rho correlation coefficients for DNA amount (ng), dimension of infected areas (mm^2^) and number of sporangia differentiated and level of significance (parenthesis). ‘-‘ Indicates no significant correlation among the variables.

#### Peroxidase activity

Grapevine peroxidases belonging to class III group and located in cell walls or vacuoles, catalyze the oxidation of a broad range of substrates, such as the cell wall protein extensin, plant growth regulators and in particular the auxin indole-3-acetic acid (IAA), and phenolics (benzoic acids, stilbenes, flavonols, cinnamyl alcohols and anthocyanins) reducing H_2_O_2_[27]. In plant-pathogen interactions, peroxidases play a role in cell wall rigidity, through a cross-linking activity on phenolic monomers that leads to the formation of suberin and the oxidative coupling of lignin subunits, and, through the hydroxylic cycle, contribute to the oxidative burst producing reactive oxygen species (ROS) that are involved in the hypersensitive response and more generally in cell death [28].

Peroxidase activity (POX) in both inoculated and not inoculated leaves was evaluated qualitatively by transmission light microscopy, and quantitatively by spectrophotometry, exploiting the chromogenic property of TMB that undergoes a change in colour upon oxidation by peroxidase in presence of hydroperoxide [29,30].

**Figure 5:**
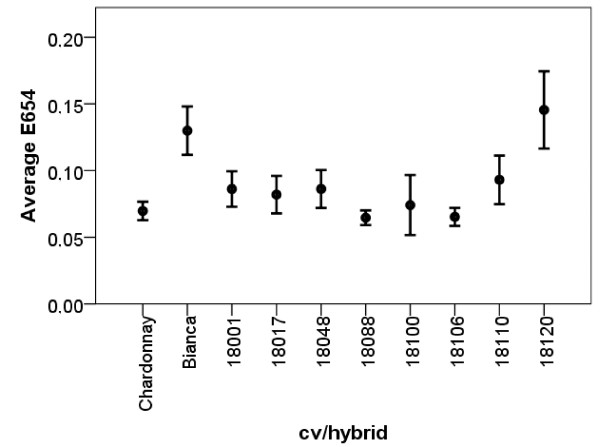
**Basal POX of the different cv/hybrids.** Bars represent standard deviation

The spectrophotometric assay indicates that, compared to the susceptible Chardonnay and the other samples tested, the resistant individuals Bianca and 18120 are characterized by a higher basal POX (Figure 5).

Wilcoxon signed-rank test showed a significant increase in POX followed the inoculation with *P. viticola* in only 38% of the cases (Table 5). Just in 4 samples (18088, 18110, 18106 and 18120) the increase in POX occurred between 1–2 dai. While in the first three cases (18088, 18110, 18106) it ranged between 0.005 and 0.014 and no significant reduction in the host colonization was observed, in the resistant hybrid 18120 the increase was more consistent (0.154) and associated with a reduced colonization of the tissues. In the other samples, including Chardonnay and Bianca, the increase occurred between 3 and 6 dai, when the pathogen was already diffusing inside the leaf tissues and differentiating the first sporangiophores. A generalised increase of POX was also detected at 4–5 dai in both resistant and suceptible genotypes by Kortekamp and Zyprian [26]. The resistant individuals Bianca and 18120 showed an higher basal POX than susceptible plants, as in the case of the resistant *V. riparia* selections Gloire de Montpellier and Lake St. George and the interspecific hybrid Seyval [26]. In these selections, the infection caused an increased production of ROS, hypersensitive response and POX in cells flanking the infection point. Apart from 18120, the early increase in POX activity was not associated with the host phenotype.

**Table 5:** **POX (ΔE) of the samples inoculated with *P. viticola *from 1 to 6 dai **

**cv/hybrid line**	**1**	**2**	**3**	**4**	**5**	**6**
Chardonnay	0.003	0.006	0.010*	0.110*	0.025	0.006
Bianca	0.058	0.014	−0.009	0.057*	−0.044	0.056*
18001	−0.019	−0.019	0.023*	0.064*	−0.020	0.016
18017	−0.030*	0.045	0.008	0.017	0.012*	0.026*
18048	−0.053	0.006	0.006	0.056*	−0.018	−0.023
18088	0.005*	−0.017*	0.011*	0.035*	0.012*	−0.009*
18100	0.006	−0.017*	0.023*	0.016	0.009*	0.009*
18106	0.033	0.009*	−0.007	0.036	0.034*	0.009*
18110	0.014*	−0.021	−0.063*	0.119*	0.033*	−0.029*
18120	−0.027	0.154*	0.003	0.032	0.120	−0.033

*Indicates a significant difference between not inoculated and inoculated samples at *P* = 0.05 level of probability.

Microscopic observations showed the occurrence of POX in all the host genotypes on the guard cell walls and vacuoles of the stomata close to the areas infected by *P. viticola* both in the early phases of the infection process, at 1–3 dai, and in the later stages corresponding to the sporangiophore differentiation, at 4–6 dai (Figure 3a,c and g). Traces of POX were also visible at the base of the sporangiophore, a structure in close contact with the stoma (Figure 3h). The susceptible cv Chardonnay did not show any blue staining inside the stomata before the beginning of sporulation (4 dai). POX, localized on both stomata and cell walls, was always associated with browning of the host cells (Figure 3c) and in the resistant individuals Bianca and 18120, with altered pathogen structures. However, it did not completely prevent the colonization of the host tissues by *P. viticola*, that was in most of the cases able to grow and sporulate (Figure 3b, f and h) even if over a reduced area. For example, 3 days after inoculation on cv Bianca POX was observed in the plasma membrane of the epidermal cells surrounding a stoma showing brown cells underneath (Figure 3c). In the area immediately under the stoma, aniline blue staining revealed an altered structure of *P. viticola* mycelium, surrounded by callose depositions and with no recognizable haustoria (Figure 3d m_1_). However, the pathogen differentiated a normal branching mycelium and haustoria with the callose neck clearly visible (Figure 3d m_2_) in the surrounding tissues. The synthesis of ROS by NADPH oxidases, balanced by detoxification systems able to maintain redox homeostasis, during different growth and reproduction phases such as hyphal growth, sporulation and spore differentiation and germination is a common feature in fungi [31]. Since the sporangia were viable, the presence of POX could be related to the differentiation of the zoospores, whereas in the case of sporangiophores, since POX is mainly located inside fungal structures in close contact with the stomata, it could be more likely a response to the oxidative stress of the host leading to the necessity of maintaining the redox balance.

#### Callose

**Figure 6:**
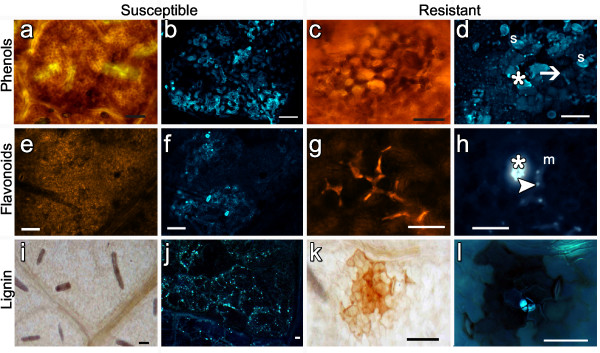
**Localization of phenolic compounds, flavonoids and lignin in infected leaf tissues.** Sensitive (a,b,e,f,i,j) and resistant (c,d,g,h,k,l) leaves have been inoculated with *P. viticola*. Phenols (a,c) and lignin (i,k) are visualized in bright field microscopy following nitrous reaction and phloroglucinol-HCl staining respectively, whereas flavonoids (e,g) and pathogen structures (b,d,f,h,j,l) in fluorescence microscopy following Wilson reaction and aniline blue staining respectively. (**a**) Phenolic compounds (red) on palisade cells of cv Chardonnay at 5 dai. (**b**) Wide diffusion of *P. viticola* mycelium inside the same tissue showed in (a). (**c**) Phenolic compounds (red-brown) inside the spongy mesophyll cells in close proximity to a failed penetration site. Hybrid Bianca at 5 dai. (**d**) Sporangia (s) and germinating zoospore (arrow) on the leaf epidermis showing fluorescent cells (asterisk), indicative of cell death. (**e**) Absence of a specific reaction to Wilson reagent in cv Chardonnay at 5 dai. (**f**) Regular diffusion of the mycelium in (e) sample. (**g**) Specific reaction to Wilson reagents (orange) inside the spongy mesophyll cells of cv Chardonnay at 5 dai. (**h**) Infected stoma covered by callose and poorly fluorescent, probably degenerating, *P. viticola* mycelium (m) with haustorial necks clearly visible in the same area showed in (g). (**i**) Presence of lignin (red-brown) inside the leaf veins of hybrid 18048 at 15 dai. (**j**) Wide diffusion of the pathogen inside sample (i). (k) Lignin on the host cell walls and inside cytoplasmic vesicles in the cells surrounding an infected stoma. Bianca at 15 dai. (**l**) Absence of mycelium, probably degenerated, inside (**k**) sample.

Callose deposition localised in the stomata, the spongy cells and around the advancing hyphae (Figures 1c,o; 3d; 6h) was the first structural response detectable in the infected tissues of cv Bianca and its siblings from 1 dai onwards. In Chardonnay, callose was limited to some stomata and detected in later phases, starting from 5 dai. Callose contributes to the penetration resistance against some agents of powdery mildew [32] and it is involved in the restriction of bacterial colonization. Callose does not seem to play an important role in limiting the development of *P. viticola* in other tolerant or resistant *Vitis* cultivars, since it is not detected until 5 dai [25,33]. In Bianca and its hybrids, on the contrary, callose deposition at the penetration points occurrs rapidly and is usually followed by the encasement of the developing hyphae and the penetrating haustoria. Callose plugging of the stomata represents a physical barrier to both the penetration and evasion of the pathogen and could be related to the reduction of sporangial amount produced in some resistant genotypes. In the host cells callose can hamper the nutrient exchange mediated by the haustorium, while its deposition around the hyphae efficiently contributes to delay or block the pathogen colonization of the host [34].

#### Phenolic compounds

Phenolics are ubiquitous compounds in plants that include numerous and diverse compounds that have a role in a large number of functions in growth, development and defence from both abiotic and biotic stresses [35]. They are mainly involved in postinfectional responses to disease agents but they also act as preinfectional barriers [32,36]. During the interaction with *P. viticola* the main phenolic compounds detected in different grapevine varieties are stilbenes, flavonoids [37-40] and phenolic compounds oxidized by POXs [41], which are accumulated more rapidly and to a higher extent in resistant genotypes in comparison to *V. vinifera*. The phenolic compounds detected in the leaves of cv Chardonnay not inoculated with *P. viticola* were mainly located in the leaf veins, whereas in Bianca they were also found in a few stomata (data not shown). Upon nitrous-acid reaction, phenolic compounds such as catechols and chlorogenic acid develop a dark cherry-red colour. Positive reactions were observed on the leaf veins, epidermis and mesophyll of resistant and susceptible plants inoculated with *P. viticola* at 5 and 7 dai, but while in susceptible tissues phenolics did not interfere with the pathogen colonization (Figure 6a), in resistant samples they concentrated inside the vacuoles of the spongy mesophyll cells immediately underneath the penetration sites (Figure 6c).

Neu's and Wilson's reagents, used for the detection of flavonoids, gave analogous results: no specific reaction to the presence of *P. viticola* was observed in susceptible plant tissues (Figure 6e), whereas the spongy mesophyll cells of the resistant hybrids showed a strong fluorescent signal and a shrinked structure (Figure 6g). Flavonoids were also detected in guard cells and on the walls of the surrounding epidermal cells.

Lignin, a complex polyphenolic compound, is a normal component of the host cell wall, particularly in the vascular system and can be generated by mechanical wounding or act as a non-degradable, mechanical barrier to fungal pathogens at a preinfectional level or in response to a pathogen attack [42,43]. Lignin was observed in both inoculated and not inoculated samples of susceptible and resistant cv/hybrids at 7 and, at higher levels, at 15 dai. It localized in the leaf veins, stomata and walls of the surrounding epidermal cells. No specific reaction associated with the pathogen presence in susceptible plants (Figure 6i,j), whereas in the resistant hybrids a strong reaction to phloroglucinol-HCl could be found inside some infected stomata (Figure 6k) where the pathogen growth was blocked at the substomatal vesicle level (Figure 6l). In the leaf tissues of the susceptible plants where phenolic compounds have been detected, the pathogen mycelium showed regular structures and a large diffusion (Figure 6b,f,j). In resistant hybrids, despite the high presence of sporangia and germinating zoospores (Figure 6d), the pathogen was not always able to colonize the tissues, since its development was either blocked at the substomatal vesicle level (Figure 6l) or it was limited to the areas close to the penetration site (Figure 6h).

## Conclusions

Durability i.e. the maintenance of an adequate level of resistance throughout the useful lifetime expected from a variety [22] is one of the most important objectives of plant breeding programmes for resistance. Useful lifetime is usually short for annual crops which can be easily replaced, but it could last several years in perennial cultivated plants, which are very costly to replace or to protect by other means against virulent pathogens. Resistant annual crops are often characterized by the presence of single resistance genes (qualitative resistance), which generally induce a strong selection pressure on the pathogen populations and, as a consequence, a rapid increase in the frequency of strains that are able to overcome the host defence reactions. Quantitative resistance due to multiple genes and resulting in reduced disease incidence is usually more useful for perennial crops, since it assures more durable results, even if adaptation to the host has also been observed in the case of polygenic traits [14]. As already pointed out, resistance to *P. viticola* is associated with the synthesis of physical barriers, such as callose [33] and lignin [37] appositions, and substances with antifungal properties such as pathogenesis-related proteins [44], peroxidases [41] and phenolic compounds [37,45]. The host response depends on many factors linked to both the environment and the pathogen. In particular, the pathogen virulence can modulate the host defence responses. Field populations of *P. viticola* that are presumably composed of different genotypes are normally used for the screening for resistance in grapevine [19,20,46], but this method prevents a comparative evaluation of the interaction between different host and pathogen genotypes. To the best of our knowledge, no prior attempt has been made to evaluate grapevine resistance as performed in the present study by combining the use of individual genotypes, isolated from single germinated oospores sampled from geographically distinct vineyards, and the quantitative assessment of the disease intensity. The results clearly show that the disease level expressed as AUDPC is determined not only by the isolate virulence and the host resistance level but also by their interaction. Therefore particularly virulent strains such as G are likely to cause conspicuous damages, at least to the leaf tissues, in hybrids characterized by a resistant behaviour against less aggressive strains. On the other hand, it must be pointed out that less severe epidemics would develop on the resistant hybrids in comparison with the susceptible varieties due to a constant reduction of the sporulation efficiency. The reduction of sporulation efficiency is of paramount importance in the field, since the amount of sporangia produced by *P. viticola* greatly affects the epidemic growth rate of the pathogen. In presence of favourable climatic conditions, in fact, even from a limited number of infection foci the pathogen can potentially spread in the host population thanks to an abundant production of sporangia, leading to severe infections on both leaves and clusters.

Histological investigations confirmed the differences in the interaction between *P. viticola* strain G and its hosts, particularly evident from 4 dai onwards. In the susceptible individuals, *P. viticola* colonization followed a regular pattern leading to an extensive growth in the host tissues and sporulation. On the contrary, the pathogen development in the resistant plants was characterized by alterations in the hyphal diameter in the early growth phases and a reduced colonization of the tissues. The resistant reactions therefore likely occurr early in the colonization process and seem to limit the pathogen growth instead of completely blocking it.

Colonization patterns in resistant and intemediate genotypes were associated with numerous changes in both the structural and chemical leaf characteristics, due to the activation of complex defense responses which eventually cause the necrotic lesions and the reduced colonization already described in Bianca [18]. A rapid callose deposition at the penetration point and around the developing hyphae seems to characterize the interaction between *P. viticola* and the resistant genotypes. The cytochemical analysis carried out in this study showed that peroxidatic activity and phenolic compounds synthesis are involved in disomogenous defense reactions of the resistant plants. In fact the pathogen colonization is stopped in the necrotic lesions but continues in the nearby tissues where no detectable host reaction occurs. Flavonoids and other phenolics were observed inside the infected stomata and surrounding epidermal cells together with shrinked and brown mesophyll cells in close contact to the developing hypha. No close association between pathogen and phenolics could be detected in the susceptible cv Chardonnay. Therefore the browning content of the cells close to the areas colonized by the pathogen in resistant plants could be the result of the oxidation of flavonoids to semiquinones and quinones, which in turn react with other phenols, amino acids or proteins forming brown products [47]. In healthy cells, the oxidases and their substrates are compartmentalized in different parts (cytoplasm and vacuoles respectively), and come in contact with each other when the cells are in some way damaged [48]. No other structural changes in the infected areas were observed until 7 dai when in the resistant genotypes lignin deposition was detected in the walls of the infected stomata and surrounding cells. In all the tested genotypes, lignin has been detected from 1 dai in the leaf veins, but its association with the pathogen occurred only in the resistant varieties in the final infection stages, as already observed by Dai and co-workers [37,49]. Lignin apposition probably represents the final stage of the defence reaction started with POX.

In conclusion, the response of Bianca and its siblings to *P. viticola* varied according to the inoculated strain, which in turn demonstrated different virulence levels. The most virulent strain, without any reduction in its overall fitness, induced the same disease intensity in Chardonnay and Bianca, suggesting that no fitness costs are associated with the resistance breakdown. However some hybrids showed an intermediate resistant phenotype and could be used in further field trials. Resistance is associated with a limitation of the pathogen growth, due to multiple reactions, ranging from callose deposition, POX activity and accumulation of phenolic compounds. From an epidemiological point of view, the reduction of sporangial production, a phenotypic trait common to to cv Bianca and all its siblings, is particularly interesting and can contribute to a more efficient downy mildew control.

## Methods

### Plant material

Fifteen grapevine hybrids (18001, 18006, 18017, 18036, 18048, 18053, 18088, 18090, 18096, 18099, 18100, 18103, 18106, 18110, 18120) obtained as described in Peterlunger et al. [50] and Bellin et al. [18], by crossing ‘Chardonnay,’ susceptible, and ‘Bianca,’ resistant, were provided by the University of Udine, Italy together with the two parental plants. Two potted plants per individual were cultivated in a screen house at the Faculty of Agriculture of the University of Milan, Italy. Actively growing plants were regularly watered *via* a drip system and fertilized with the controlled release fertilizer Osmocote Pro (Scotts Professional Italia Srl, Treviso, Italy). Both propagation and experimental inoculations with *P. viticola* were carried out on the 3^rd^-4^th^ fully expanded leaf starting from the apex of the shoot. Leaves or leaf discs were placed, lower surface upwards, in Petri dishes (Ø 9 or 6 cm) containing WAK medium: 1% water-agar (Agar Bacto, Oxoid) amended with 2 mg/L kinetin (6-Furfurylaminopurine, Sigma-Aldrich s.r.l. Italy), to retard leaf senescence [51]. The samples were incubated in a growth chamber at 20 ± 2°C (16 h of light per day).

### *P. viticola* isolates

Five *P. viticola* isolates, weekly propagated on detached leaves of cv ‘Cabernet franc’ plants grown in screen house, were considered in this study. The strains, BAI, C, G, SO, and M, were isolated from single germinating oospores differentiated respectively on cv ‘Baresana’ (Puglia, southern Italy), cv ‘Corvina’, cv Merlot (Veneto, north-eastern Italy), cv ‘Nebbiolo’ and cv 'Pinot gris’ (Lombardia, northern Italy).

Grapevine leaves showing mosaic symptoms were sampled in October in order to collect leaf fragments rich in oospores which were overwintered in controlled conditions of temperature and humidity (BAI, C, G and M) or in vineyard (SO) until April. Germination assays were carried out on the oospores isolated from the leaves and incubated for one week on 1% water-agar (Agar Noble, DIFCO) at 20°C [52]. Single germinating oospores with fully developed macrosporangia were transferred with a sterile needle to 10 μL water droplets on leaf discs (Ø 1.5 cm) of cv ‘Cabernet franc’. At the end of the incubation period, sporangia were propagated on detached leaves as described for the leaf disc assay in the following paragraph.

### Experimental inoculations and evaluation of the disease severity

The experimental inoculations were carried out on three replicates of 4 leaf discs per plant placed on WAK in Petri dishes (Ø 6 cm) by uniformly spraying 1 mL of a sporangial suspension (5x10^4^ sporangia/mL) per dish. The disease severity on the samples incubated in growth chamber (20 ± 2°C, 16 h light) was evaluated at the stereo microscope (Leica Wild M10) every 24 hours between 4 and 9 days after inoculation (dai). The leaf discs were classified in eight classes (0–7) according to the percent area showing sporulation, where 0 = absence of sporulation, 1 = 0.1-2.5%, 2 = 2.5-5%, 3 = 5–10%, 4 = 10–25%, 5 = 25–50%, 6 = 50–75%, and 7 = 75–100% of the leaf area covered by sporulation. The percentage of sporulating area (PSA) of each replicate was calculated from the modified formula of Towsend and Heuberger [53] taking into account the sporulation classes: $PSA=\frac{\sum \left(n\times v\right)}{7N}\times 100$ where *n* = number of leaf discs in each class, *v* = numerical value of each class and *N* = total number of leaf discs in the sample. Area under disease progress curve (AUDPC) was calculated [54,55] from PSA values in order to evaluate the host phenotype and virulence of *P. viticola* strains. AUDPC was chosen because it summarizes disease intensity over time providing, at the same time, a quantitative measure of the pathogen virulence [55-57].

### Quantification of sporangia and leaf tissue colonization

The samples inoculated with the *P. viticola* isolate G were used to count the number of sporangia differentiated, to measure the colonized leaf areas and to quantify the pathogen DNA in each grapevine line at 9 dai. Sporangia were detached from the sporangiophores by vortexing each leaf disc in a 1.5 mL tube containing 500 μL of distilled water. The average number of sporangia (SN) per leaf disc was calculated from the average number of sporangia per mL of suspension determined by counting the spores in two replicates of 10 μL of sporangial suspension in a Neubauer counting chamber (Riechert Bright-Line haemocytometer, Hausser Scientific, Horsham, PA, USA) under a optical bright field microscope (Leitz Orthoplan). Sporangia were checked for viability by germination assays [58].

Immediately after the release of the sporangia from the leaf surface, the discs were fixed and cleared in 75% ethanol and kept at 4°C. The samples were soaked in distilled water for 3 times, stained with 0.05% aniline blue in 0.067 M K_2_HPO_4_ (pH 9) for 24 hours and observed under a fluorescence microscope (Olympus BX50 equipped with a Q-Imaging Retiga 2000R digital camera; DAPI filter λexc 340–380 nm, dichroic mirror 400 nm, barrier filter 435–485 nm). The reaction of aniline blue with β-1,3-glucans, makes the fungal structures of *P. viticola* visible under UV light [59]. Pictures of the leaf discs colonized by the pathogen were captured at 4x magnification to measure the infected areas (IA). The areas in mm^2^ were estimated from the corresponding number of pixels measured with Adobe Photoshop 7.0 software (Adobe Systems Incorporated, San Jose, CA, USA).

### *P. viticola* DNA quantification

The mycelial growth of *P. viticola* strain G in the host tissues was estimated from the quantification of the pathogen DNA isolated from the leaf discs by using primers specific for the ITS (Internal Transcribed Spacer) region of the nuclear ribosomal DNA (rDNA) of *P. viticola*.

The leaf discs used for the colonization assessment under fluorescence microscope were rinsed in double distilled sterile water, ground in liquid nitrogen with the aid of mortar and pestle. The DNA, extracted following the protocol described by Toffolatti et al. [52], was resuspended in 40 μL of double distilled sterile water. DNA from *P. viticola* sporangia (2 × 10^5^ sporangia) was used as a positive control and DNA from uninfected leaves as a negative control. DNA quality and quantity were spectrophotometrically checked (NanoDrop Technologies, Wilmington, DE). *P. viticola* DNA was serially diluted (40, 10, 1, 0.1, 0.01, 0.001, 0.0001 ng/μL) in water for the standard curve, or in 1 ng/μL *V. vinifera* DNA, to investigate possible influences of the host DNA on the amplification of the pathogen DNA.

*P. viticola* primer sequences specific for the region between the internal transcribed spacer 1 (ITS 1) and the 5.8 S in the ITS sequence were used for the absolute quantification of the pathogen DNA by real-time PCR and Taqman chemistry as described by Valsesia et al. [60]. Reactions were performed in 30 μL aliquots with 1x RealMasterMix probe + ROX (5 Prime GmbH, Hamburg, Germany), 900 nM forward (TCCTGCAATTCGCATTACGT) and reverse (GGTTGCAGCTAATGGATTCCTA) primers, 250 nM 5’FAM-labeled probe quenched by 3’Tamra (TCGCAGTTCGCAGCGTTCTTCA), and 5 μL of DNA template. *P. viticola* and *V. vinifera* DNA standards ranged from 0.005 to 200 ng. Real-time PCR was performed by the Applied Biosystems 7300 Real-Time PCR System in MicroAmp optical 96-well plates sealed with optical adhesive covers (Life Technologies Italia, Monza, Italy). Thermal conditions consisted of 2 min at 50°C, 10 min at 95°C and 40 cycles of 95°C for 15 sec and 60°C for 1 min. Fluorescence emission was detected at the annealing/extension step (60°C) and ROX was used as passive reference dye. Each PCR assay included negative controls (water and 1 ng/μL *V. vinifera* DNA) and *P. viticola* DNA standards. Each reaction was run in duplicate. Cqs (Cq = cycle number at which the fluorescence generated within a reaction crosses the threshold) were automatically calculated by the SDS software on the 7300 Real-Time PCR System. The standard curve was constructed by plotting Cqs against the logarithmic values of *P. viticola* DNA (ng): the correspondent DNA in the unknown samples (X_0_) was calculated after linear regression analysis as X_0_ = 10^(Ct-intercept)/slope^ whereas the PCR efficiency (E) was calculated as E = 10^-1/slope^-1 [61].

### Histological analyses

Young leaves detached from both the parental varieties and the corresponding hybrids were placed on WAK in Petri dishes, inoculated with numerous 10 μL droplets of isolate G sporangial suspension and incubated as previously described. An analogous procedure was carried out using double-distilled, sterile water on the control leaves. The leaf areas under the droplets were collected from the inoculated and not-inoculated samples with a cork borer (Ø 0.5 cm) and kept in 75% ethanol at 4°C. Both the callose deposition by the plant and the pathogen structures were investigated using aniline blue staining at 1, 2, 3, 4 and 7 dai. Hyphal length and diameter were measured at 2 and 2–4 dai respectively by analysing the images with Bel View software (BEL Engineering srl, Monza, Italy). The results were expressed as average values of three independent measures of three hyphae.

Nitrous acid reaction [62] was used for the detection of phenolic compounds between 1 and 7 dai, phloroglucinol-HCl [37,63] for lignin at 7 and 15 dai and Neu’s and Wilson’s reagents for flavonoids at 5 and 10 dai [37]. The samples were mounted in 75% glycerol on glass slides and observed under bright field light microscopy (Olympus BX50), for phenols and lignin detection, or under UV light (Nikon Eclipse 80i equipped with a video-confocal system; Nikon Instruments S.p.a., Calenzano, FI, Italy) with DAPI filter, after staining for callose and flavonoids. The presence of autofluorescence was evaluated on unstained samples.

The association between the above mentioned defence metabolites and *P. viticola* structures was assessed by staining with aniline blue as described for the leaf tissues colonization. The pathogen structures were visualized in red, using DAPI filter, and the leaf tissues in green, using a FITC filter (ex 465–495 nm, dm 505 nm, ba 515–555).

Histochemical visualization of POX under light microscopy was achieved by incubating the fixed leaf discs for 10 min in a medium containing 10 mg TMB (3,3’,5,5’-Tetramethylbenzidine), 2.5 mL ethanol, 50 mL acetic acid/sodium acetate buffer solution pH 4.5, 3 mL 3% H_2_O_2_ and 47,5 mL H_2_O [37]. The association of POX with the structures of *P. viticola* was confirmed by aniline blue staining of the tissues. Moreover, cytosolic POX was assessed by a spectrophotometric assay based on peroxidase reaction with hydrogen peroxide, using TMB as chromogenic substrate as described by Kortekamp and Zyprian [26]. The assay was carried out on two leaves of parental cultivars (Chardonnay and Bianca) and selected hybrid lines (18048, 18088, 18100, 18106, 18110 and 18120) inoculated with isolate G or distilled, sterile water. Six leaf discs were sampled per leaf and the assay was repeated twice. Basal POX was estimated from the extinction coefficients of the samples inoculated with water. The involvement of peroxidatic activity in grapevine-*P. viticola* interaction was evaluated taking into account ΔE, *i.e.* the difference between the spectrophotometric values obtained from the samples inoculated with the pathogen and those assessed in the uninoculated control leaves.

### Statistical analyses

Upon satisfaction of the requirements for analysis of variance, AUDPC values were compared using one-way ANOVA and multiple comparison of the means (REGW-F test). A combined analysis of variance (two-factors ANOVA) was also carried out to test the interaction between strains and cv/hybrids (fixed factors).

Only for *P. viticola* strain G, each leaf disc was classified according to the average value of the class used to evaluate the disease severity in order to estimate the Gompertz growth model parameters by linear method on three replicates [64]. The pathogen fitness (PF) on each host genotype was evaluated from a composite index using a modified method of Flier and Turkensteen [65]: $PF=\frac{IEI\times SPOR}{T_{10}\times MGR}\times 10^{3}$ where IEI is the infection efficiency index, calculated as the fitted asymptotic value of disease severity at 10 dai; SPOR is the natural logarithm of the average number of sporangia per cm^2^ of infected leaf area; T_10_ is the time expressed in hours required for severity to reach 10%; MGR is maximal curve growth rate, calculated as the first derivative of the fitted curve at the point of inflection; and 10^3^ is used for scaling purpose. Comparison of the mean values of the fitness components was carried out by non parametric statistics using Kruskal-Wallis test on the data transformed in ranks. ANOVA and multiple comparison of the mean PF values (REGW-F test) were performed to evaluate significant differences between the different grapevine varieties.

The existence of linear correlation among the amount of DNA (ng), sporangia and mm^2^ of infected area of each leaf disc inside the different grapevine lines at 7 dai was assessed by non parametric statistics Spearman’s rho at the 0.05 level of significance.

The potential increase in POX as a response to the interaction with *P. viticola* was estimated by Wilcoxon signed-rank test for data pairs on the extinction coefficients of inoculated and not inoculated samples.

Statistical analysis was carried out with the SPSS Statistics 18.0 software.

## Abbreviations

AUDPC: Area under disease progress curve; dai: Days after inoculation; DAPI: 4’,6-diamidino-2-phenylindole; IA: Infected Area; IEI: Infection efficiency index; IR: Intermediate-resistant phenotype; IS: Intermediate-susceptible phenotype; MGR: Maximal growth rate; PF: Pathogen fitness; POX: Peroxidase activity; PSA: Percentage sporulating area index; QTL: Quantitative Trait Loci; SPOR: The natural logarithm of the average number of sporangia per cm^2^ of infected leaf area; T_10_: Time expressed in hours required for disease severity to reach 10%; TMB: 3,3’,5,5’- Tetramethylbenzidine; V: Variable phenotype; WAK: Water Agar Kinetin medium.

## Competing interests

The authors declare that they have no competing interests.

## Authors’ contributions

ST carried out all the studies and drafted the manuscript, GV participated in the statistical analysis of the data and helped to draft the manuscript, DM helped to carry out microscopic examinations, AV conceived the study, participated in all steps of the analysis and drafted the manuscript. All authors read and approved the final manuscript.
